# Clinical effectiveness and predictors of response to topiramate plus lifestyle modification in youth with obesity seen in a weight management clinical setting

**DOI:** 10.3389/fendo.2024.1369270

**Published:** 2024-05-10

**Authors:** Eric M. Bomberg, Justin Clark, Kyle D. Rudser, Amy C. Gross, Aaron S. Kelly, Claudia K. Fox

**Affiliations:** ^1^Center for Pediatric Obesity Medicine, Department of Pediatrics, University of Minnesota Medical School, Minneapolis, MN, United States; ^2^Division of Endocrinology, Department of Pediatrics, University of Minnesota Medical School, Minneapolis, MN, United States; ^3^Division of Biostatistics and Health Data Science, School of Public Health, University of Minnesota, Minneapolis, MN, United States

**Keywords:** anti-obesity agents, obesity, pediatric obesity, obesity management, topiramate

## Abstract

**Introduction:**

Obesity affects approximately 20% of U.S. youth. Anti-obesity medications (AOMs) are promising lifestyle modification adjuncts for obesity treatment, and topiramate is commonly prescribed in pediatric weight management clinics. It is important to determine “real-world” effectiveness of AOMs and, given shifts towards personalized approaches, characteristics potentially predicting better or worse response. We therefore sought to describe clinical effectiveness from topiramate plus lifestyle modification, and to determine if baseline phenotypic characteristics are associated with better or worse response.

**Methods:**

We performed a retrospective cohort study (2012-2020) among youth (<18 years old) followed in a U.S. academic-based weight management clinic. Baseline characteristics (i.e., body mass index (BMI), liver function tests, eating-related behaviors) and outcomes (%BMI of 95^th^ percentile (%BMIp95), BMI, percent %BMI change, weight) were determined through review of electronic health records and clinic intake survey data.

**Results:**

Among 282 youth prescribed topiramate plus lifestyle modifications (mean baseline age 12.7 years, %BMIp95 144%), %BMIp95 and percent BMI change were statistically significantly reduced at each time point (1.5-, 3-, 6-, and 12-month %BMIp95 reductions: -2.2, -3.9, -6.6, and -9.3 percentage points, respectively; percent BMI reduction: -1.2%, -1.9%, -3.2%, and -3.4%, respectively; all p<0.01). Considering multiple comparisons, no baseline characteristics statistically significantly predicted response at any time point.

**Conclusions:**

We found that topiramate plus lifestyle modification reduced %BMIp95 and BMI among youth in a weight management clinical setting, and that no baseline characteristics evaluated were associated with response. These results should be considered preliminary given the observational nature of this study, and prospective studies are needed to further characterize clinical effectiveness and identify and confirm potential predictors of response.

## Introduction

Pediatric obesity (BMI ≥95th percentile) currently affects around 20% of U.S. youth (1). As lifestyle modification therapy alone often fails to result in clinically significant and durable weight loss (e.g., Danielsson et al. showed that 2% of adolescents with severe obesity [BMI ≥ 1.2 times the 95th percentile] receiving 3 years of lifestyle modification experienced significant weight loss) (2), anti-obesity medications (AOMs) have become promising adjuncts and are being prescribed with increasing frequency (3). While AOMs combined with lifestyle modification have overall been associated with BMI reduction, few studies have evaluated their effectiveness among youth in clinical settings. This is partly due to historically lower utilization resulting from overall low availability of weight management providers, the relative newness of U.S. Food and Drug Administration (FDA)–approved options, and accessibility issues, among other reasons (3, 4). Further, studies evaluating nearly every AOM in conjunction with lifestyle modifications have shown substantial individual-level variability in effectiveness, with some losing significant amounts of weight and others not, or even gaining weight while on treatment (5). As pediatric obesity is associated with increased risk for the development of numerous obesity-related health sequelae (6–9), identifying “real-world” clinical effectiveness and person-specific characteristics that may predict response to AOMs plus lifestyle modifications is critical (10). Doing so may help determine what responses may be expected in clinical settings and those who may be more or less likely to benefit from specific AOMs (10).

While most U.S. FDA-approved AOMs only gained pediatric approval in the last couple of years (since 2021), several medications have been used off-label for weight management for longer (11). Of these, topiramate has perhaps been the most commonly prescribed in pediatric weight management clinics (3). Topiramate was U.S. FDA-approved for the treatment of seizures in youth 2–16 years of age, and for the prevention of migraine headaches in 12–17 year-old adolescents, in 1999 and 2014, respectively; however, has not been FDA or European Medicines Agency (EMA) approved for the treatment of obesity in either children or adults. Its central purported mechanisms for weight loss include increasing gamma-aminobutyric acid (GABA) and decreasing glutamate and dopamine secretion, subsequently decreasing appetite, food cravings, and binge eating; and through carbonic anhydrase inhibition, which can alter taste of carbonated beverages (12). Several studies have explored topiramate for weight loss in adults, showing mean weight loss around 5%–7% after 6–12 months on doses around 100 mg daily (13–18). In these studies, the individual weight loss response to topiramate was highly variable as seen by the fact that all reporting standard deviations showed these to be as great or greater than mean weight loss values (13–18).

Despite the fact that topiramate is commonly prescribed in pediatric weight management clinics, little is known about its effectiveness in this clinical setting, or about potential person-specific characteristics that may predict those more or less likely to experience benefit. Our group previously showed a mean 6-month 4.9% BMI reduction among 28 adolescents prescribed topiramate plus lifestyle modification in a pediatric weight management clinic (19). While substantial individual-level variability in response was observed, given the small sample size baseline characteristics associated with response could not be evaluated (19). Other studies have also shown overall BMI reduction or stabilization, and that baseline characteristics including higher BMI and younger age may predict response (20–24). However, these studies included both youth and adults with or without obesity, those not necessarily followed in weight management clinics, those not prescribed topiramate for obesity treatment (i.e., prescribed for epilepsy, psychiatric disorders, etc.), and/or those with known genetic syndromes (i.e., Prader-Willi) (20–24).

Determining effectiveness of AOMs plus lifestyle modifications in clinical settings is important, as this may differ compared to outcomes seen in clinical trials that historically have enrolled highly selected populations that may not adequately represent those seeking medical care more broadly. Potential reasons for these differences include generally better compliance in clinical trials involving obesity interventions, and that participants in such trials tend to be primarily white, female, and have minimal co-morbidities (i.e., no significant depression and/or anxiety, hemoglobin A1c ≤10%, etc.), often not the case in clinical settings (25).

Our goals in this retrospective cohort study among youth receiving care in a pediatric weight management clinic were to (1) evaluate effectiveness of topiramate plus lifestyle modification in a clinical setting and (2) identify potential baseline phenotypic characteristics that may predict better or worse response to this intervention. We hypothesized that baseline characteristics including sex; age; obesity classification (class 1 vs. classes 2 and 3); presence of abnormal liver transaminases [aspartate aminotransferase (AST), alanine aminotransferase (ALT)]; elevated hemoglobin A1c; depression, anxiety, hunger, and binge eating tendencies; and additional eating-related behaviors would be associated with topiramate plus lifestyle modification response 1.5, 3, 6, and 12 months after initiation.

## Materials and methods

### Study design and participants

This was a retrospective cohort study performed through review of electronic health record (EHR) and baseline clinic intake survey data among children and adolescents receiving care in a U.S., Midwestern, academic health center–based pediatric weight management clinic from February 2012 through December 2020. All patients seen in the clinic receive lifestyle modification therapy as part of management, including nutrition (i.e., visits with registered dieticians) and exercise counseling supported by behavioral modification strategies.

Participants included youth seen in the weight management clinic who did not opt out of having their EHR reviewed for the purposes of research via the Consent for Services form that all patients and/or caretakers complete. In a recent study using EHR data from patients treated in our clinic, the prevalence of youth and/or caretakers on their behalf opting out of research was ~1% and was not meaningfully different by race/ethnicity (26). Additional inclusion criteria included age <18 years old when topiramate first prescribed, treated in the clinic for ≥1.5 months after this time, and ≥1 follow-up visit during the study period.

Exclusion criteria included the following: history of metabolic/bariatric surgery; obesity associated with a known genetic disorder (i.e., melanocortin 4 receptor mutation, leptin deficiency) or syndrome (i.e., Prader-Willi, Alstrom syndrome); known hypothalamic dysfunction (i.e., history of craniopharyngioma, optic glioma, brain irradiation); hyperthyroidism or uncontrolled hypothyroidism (as evidenced by thyroid stimulation hormone ≥10 mIU/L during the study period); started and/or altered doses of other medications associated with weight gain (i.e., systemic steroids, atypical antipsychotics, insulin) or loss (i.e., other AOMs, stimulants for attention-deficit hyperactivity disorder) within 6 months before the study period (3 months for selective serotonin reuptake inhibitors and serotonin and norepinephrine reuptake inhibitors); and unhealthy weight control behaviors (i.e., history of vomiting foods, laxative use for weight loss). If a patient was started on additional medication(s) for obesity treatment (i.e., initially started on topiramate, 3 months later phentermine was added), only data from prior to additional medication(s) being initiated were included in analyses given the goal of assessing topiramate plus lifestyle modification response. Further, if other weight-altering medication(s) were adjusted (e.g., dose increased/decreased), data from beyond that time was also censored for similar reasons. This study was approved by the university’s institutional review board.

### Variables

Prescribed topiramate dosages were obtained from EHR medication lists. Mean doses at each time point were computed as the average over the entire course in those whose data went out to that point. For example, the average [standard deviation (*SD*)] topiramate dose among patients whose furthest data availability was 6 months was 36.4 ± 19.8 mg (see **Supplementary Table S1**). Many patients prescribed topiramate in our clinics are started on 25 mg daily for the first week, increasing to 50 mg for the second week, and then 75 mg until seen again in clinic, at which time further dosing changes may or may not occur depending upon response. This dosing generally aligns with that reported in several studies and is close to that included in high-dose phentermine/topiramate (92 mg of topiramate) (13–18, 27). Younger patients may be started on lower doses (e.g., 25–50 mg), and all medication-based decisions are made at the discretion of the prescribing provider, and with shared decision making involving patients and their families.

Baseline BMI percent of the 95th percentile (%BMI95), BMI (kg/m^2^), and weight (kg) were selected as those values recorded closest to the initial topiramate order. Notably, while most patients had these recorded at the visit they were first prescribed topiramate, due to the COVID-19 pandemic we also included those with measurements obtained within 2 weeks before or after the initial prescription. Weight-related outcomes were determined at the following time points: (1) 1.5 months (any visit 4–8 weeks after initial prescription), (2) 3 months (any visit 9–15 weeks after initial prescription), (3) 6 months (any visit 20–28 weeks after initial prescription), and (4) 12 months (any visit 44–60 weeks after initial prescription). We chose to increase the allowable encounter window for later visits as weight loss is generally nonlinear [often greater earlier in courses as seen in AOM clinical trials (28–30)], and given the practical consideration that follow-up over time will occur more sporadically. If more than one anthropometric measure was available within the above time points, they were averaged.

Baseline phenotypic characteristics hypothesized to predict better or worse weight loss response to topiramate plus lifestyle modifications were selected *a priori* based on previous literature and/or biologic plausibility (see **Table 1**) (2, 5, 12, 31–45). For example, sex was chosen as studies have shown that topiramate may significantly reduce leptin levels in females and serum leptin levels are higher in females compared to males (31–33). We chose to examine baseline characteristics that may be positively or negatively associated with response as both may be clinically meaningful.

**Table 1:** Hypothesized predictors of weight loss response.

PREDICTOR OF RESPONSE	RATIONALE	HYPOTHESES
**Sex**	Topiramate found to significantly reduce leptin levels in female rats; serum leptin levels higher in females than males (31–33)	Females (vs. males) = ↑ Response^a^
**Age**	Oral topiramate clearance decreases with age until puberty (34, 35); older/younger age both associated with increased weight loss to AOMs among youth in separate studies (2, 36)	↑ or ↓ = ↑ Response^a^
**Obesity category** (Class 1–3)	Higher BMI associated with increased weight loss among youth/adults on topiramate for epilepsy (20, 37); associated with increased weight loss to several obesity interventions (5)	↑ Obesity category = ↑ Response^a^
**Liver transaminases** (AST/ALT)	~20% topiramate metabolized in liver; clearance reduced ~26% in patients with moderate/severe hepatic impairment (38, 39)	Presence (vs. absence) = ↓ Response^a^
**Hemoglobin A1c**	GABA (produced at high levels in *beta* cells) may counteract insulin’s effects (40, 41)	↑ = ↓ response^a^
**Depressive symptoms**	New/worsening depression (associated with exacerbating obesity in adolescents (42)) reported side effects	Presence (vs. absence) = ↓ Response^a^
**Anxiety symptoms**	New/worsening anxiety (associated with exacerbating obesity in adolescents (43)) reported side effects	Presence (vs. absence) = ↓ Response^a^
**General hunger^b^**	Topiramate modulates GABA, glutamate, and dopamine (12); high hunger among most consistent predictors of weight loss response to AOMs (5)	Presence (vs. absence) = ↑ Response^a^
**Binge eating tendencies^c^**	Studies in adults with binge eating disorder and obesity show short-term efficacy with topiramate (44)	Presence (vs. absence) = ↑ Response^a^
**Nighttime eating^d^**	Topiramate modulates GABA, glutamate, and dopamine (12)	Presence (vs. absence) = ↑ Response^a^
**Food responsiveness^e^**	Topiramate modulates GABA, glutamate, and dopamine (12)	Presence (vs. absence) = ↑ Response^a^
**Emotional over-eating^e^**	Emotional eating associated with anxiety (see “Anxiety” above) (45)	Presence (vs. absence) = ↓ Response^a^
**Enjoyment of food^e^**	Topiramate modulates GABA, glutamate, and dopamine (12)	Presence (vs. absence) = ↑ Response^a^
**Satiety responsiveness^e^**	Topiramate modulates GABA, glutamate, and dopamine (12)	Presence (vs. absence) = ↓ Response^a^

AOM, anti-obesity medication; ALT, alanine aminotransferase; AST, aspartate aminotransferase; BMI, body mass index; CYP, Cytochrome P450; FDA, Food and Drug Administration; GABA, gamma aminobutyric acid.

^a^Response including percent BMI of 95th percentile (%BMIp95), BMI (kg/m^2^), weight (kg). ^b^Assessed via question: “I am hungry all the time,” scaled never (0 times/week), rarely (1–2 times/week), sometimes (3–4 times/week), and often (>5 times/week). Considered present in those responding rarely, sometimes, or often. ^c^Assessed via questions: “I binge on food (i.e., I eat a large amount of food in a short period of time, like 2h)” and “I feel ‘out of control’ when I eat,” both scaled never (0 times/week), rarely (1–2 times/week), sometimes (3–4 times/week), and often (>5 times/week). Considered present in those responding rarely, sometimes, or often to both questions. ^d^Assessed via question: “Wakes up and eats in the middle of the night,” scaled never (0 times/week), rarely (1–2 times/week), sometimes (3–4 times/week), and often (>5 times/week). Considered present in those responding rarely, sometimes, or often. ^e^Assessed via Child Eating Behavior Questionnaire; items scored 1–5 (higher scores indicate higher intensity of specific eating behavior).

For outcomes analyses, the primary assessment used for response was change in %BMIp95 percentage points from baseline to each time point (46, 47). Of note, the number of participants at each time point do not represent only those still being followed in the pediatric weight management clinic by that respective time point. Rather, they represent those with data available at that time point, including within a reasonable window for data interpolation as mentioned above (i.e., for the 6-month time point, had data available 20–28 weeks after initial prescription). For example, it is possible for a patient to have data available at the 3- and 12-month time points, however, not at 6 months, and also that patients may be seen less frequently later in their course due to clinical success (e.g., not needing to be seen as often), thereby leading to sparser data available at later time points. Further, outcomes at each time point may be missing for other reasons, particularly the rise in virtual visits since the COVID-19 pandemic inception (i.e., only weight and not height available in a virtual visit). We therefore additionally examined reasons for missing outcomes data, including losses to follow-up, no data within analysis window, topiramate discontinued by provider, and data censored due to additional weight-altering medication(s) (including non-weight management related) started or doses adjusted within analyses windows.

As for predictors of response, sex, age group (10.0–11.9, 12.0–15.0, and >15.0 years old), obesity classification [class 1 obesity = %BMIp95 1.0–1.19 times 95th percentile; class 2 obesity = %BMIp95: 1.2–1.39 times the 95th percentile; class 3 obesity = %BMIp95 ≥1.4 times the 95th percentile as per American Academy of Pediatrics Clinical Practice Guidelines (47)], and presence of abnormal AST or ALT and/or elevated hemoglobin A1c were derived from EHR data review.

Patients presenting to our clinics for initial evaluation are also instructed to complete baseline intake surveys that include questions regarding binge eating tendencies, general hunger, and nighttime eating; depression [via Patient Health Questionnaire–9 (PHQ-9) (48)]; anxiety [via Generalized Anxiety Disorder–7 (GAD-7) (49)]; and the Child Eating Behavior Questionnaire (CEBQ) including assessments for the following eating-related behaviors: food responsiveness, emotional over-eating, enjoyment of food, and satiety responsiveness; validated in previous studies) (50–52). Depressive symptoms were considered present if PHQ-9 score ≥5 (indicating mild depressive symptoms or higher), and anxiety symptoms considered present if GAD-7 score ≥10 (indicating moderate or severe anxiety) (48, 49).

Binge eating tendencies were assessed with the following 2 questions: “I binge on food (i.e., I eat a large amount of food in a short period of time, like 2h)” and “I feel ‘out of control’ when I eat,” both scaled never (0 times/week), rarely (1–2 times/week), sometimes (3–4 times/week), and often (>5 times/week). Binge eating tendencies were considered present in those responding rarely, sometimes, or often to both questions. General hunger was assessed with the question “I am hungry all the time,” scaled never (0 times/week), rarely (1–2 times/week), sometimes (3–4 times/week), and often (>5 times/week). Nighttime eating was assessed with the question “Wakes up and eats in the middle of the night,” scaled never (0 times/week), rarely (1–2 times/week), sometimes (3–4 times/week), and often (>5 times/week). General hunger and nighttime eating were considered present in those responding rarely, sometimes, or often, respectively.

Intake surveys, only available in English until 2021 (Spanish since added), were mailed out prior to initial clinic visits, and youth/families were instructed to complete and bring them to this appointment. We also evaluated the prevalence of youth from non-English speaking families in our sample, as they may not have received the baseline intake survey. Given several additional potential reasons as to why intake survey data may be unavailable for analysis (i.e., no time to complete survey prior to appointment, forgot at home), we compared survey completers and non-completers by insurance type (pubic vs. private) as a surrogate marker for social determinants of health (53).

### Statistical methods

Baseline characteristics are presented as descriptive summaries including mean and *SD* for continuous variables and frequency with percent for categorical variables. Changes in weight-related outcomes (%BMIp95, BMI, weight) from baseline to pre-specified time points were assessed via *t*-tests, and for each specific time point only patients with data available at that time were included in analyses. Associations between baseline characteristics and weight-related outcomes were determined via separate univariate analyses, regressing percentage point change in %BMIp95 to a given time point on the baseline characteristic.

Confidence intervals and *p*-values for regression analyses were based on robust variance estimation. For missing values within the specific date windows due to inconsistent follow-up occurring in the clinical setting, linear interpolation using the most recent measure(s) prior to or closest after a target time point were utilized to compute an imputed BMI. Statistical significance was based on a two-sided type I error rate of 0.05 (*p* < 0.05); however, to account for multiple comparisons, we also applied the Holm method (54, 55). All statistical analyses were performed using R version 3.6.0 (R Foundation for Statistical Computing, Vienna, Austria).

## Results

We identified 282 youth [61% female; mean baseline age 12.7 years (range: 3.51–17.98 years old); BMI = 36.5 kg/m^2^; %BMIp95 144 (average in the class 3 pediatric obesity category)] prescribed topiramate plus lifestyle modification and meeting additional study criteria (see **Table 2** for baseline descriptive variables). The mean dose was largely stable over time (see **Supplementary Table S1**), ranging from 34.2 ± 20.8 mg [at the 1.5-month time point (269 patients with data available)] to 39.7 ± 19.0 mg (at the 12-month time point [59 patients with data available]. Forty-two percent and 44% of patients had baseline AST and ALT tests available, of which 10% and 18% had abnormalities (elevations) in these, respectively. Seventeen percent to 32% completed various elements of the clinic intake survey and had this data available for analyses. Fifteen percent of youth were from non-primary English-speaking families. We found no statistically significant differences in those completing versus not completing clinic intake surveys by insurance type (*p* = 0.14). Among patients completing intake surveys, presence of baseline binge eating tendencies, nighttime eating, and meeting criteria for depressive and/or anxiety symptoms were present in 19%–34%, while general hunger was present in 69% (see **Table 2**).

**Table 2:** Baseline descriptive statistics for 282 youth prescribed topiramate plus lifestyle modification therapy in a pediatric weight management clinic (February 2012–December 2020).

	# Patients with data available	Value
Sex		
Female (*n*, %)	282	172 (61%)
Anthropometrics		
Age, years (mean, *SD*)	282	12.7 ± 3.1
Weight, kg (mean, *SD*)	282	91.1 ± 28.9
BMI, kg/m^2^ (mean, *SD*)	282	36.5 ± 7.6
%BMIp95 (mean, *SD*)	282	144 ± 26
Insurance type		
Public	227	108 (48%)
Private	227	119 (52%)
Liver function tests: AST		
Liver function: AST (mean, *SD*)	119	28.8 ± 27.2
Presence of abnormal AST based on lab value (*n*, %)	119	12 (10%)
Liver function tests: ALT		
Liver function: ALT (mean, *SD*)	125	42.9 ± 52.9
Presence of abnormal ALT based on lab value (*n*, %)	125	23 (18%)
Glycemic status		
Hemoglobin A1c (mean, *SD*)	124	5.4 ± 0.4
Eating behaviors and co-morbid psychiatric diagnoses		
Presence of binge eating tendencies^a^ (*n*, %)	50	16 (32%)
Presence of general hunger^a^ (*n*, %)	49	34 (69%)
Presence of nighttime eating^a^ (*n*, %)	50	17 (34%)
Met criteria for depression^b^ (*n*, %)	47	12 (26%)
Met criteria for anxiety^c^ (*n*, %)	47	9 (19%)
Childhood eating behavior questionnaire scores^d^		
Food responsiveness (mean, *SD*)	87	3.5 ± 1.2
Emotional over-eating (mean, *SD*)	89	2.8 ± 1.0
Enjoyment of food (mean, *SD*)	88	4.1 ± 0.8
Satiety responsiveness (mean, *SD*)	87	2.1 ± 0.6

ALT, alanine aminotransferase; AST, aspartate aminotransferase; BMI, body mass index; %BMIp95, BMI percent of the 95th percentile; Reference Ranges AST: 3–11 years old, 0 mg–50 mg/dl; 12–19 years old, 0–35 mg/dl; ≥ 20 years old: 0–45 mg/dl); Reference Ranges ALT: 0–19 years old, 0 mg–50 mg/dl; ≥ 20 years old male, 0–70 mg/dl; ≥ 20 years old female, 0–50 mg/dl; ^a^Per clinic intake survey questions; ^b^Met criteria for depression based on Patient Health Questionnaire–9 score ≥5 indicating mild depressive symptoms or higher; ^c^Met criteria for anxiety based on Generalized Anxiety Disorder–7 score ≥ 10 indicating moderate or severe anxiety symptoms; ^d^Childhood Eating Behavior Questionnaire Scores: items score 1–5 (higher scores indicate higher intensity of specific eating behavior).

**Figure 1:**
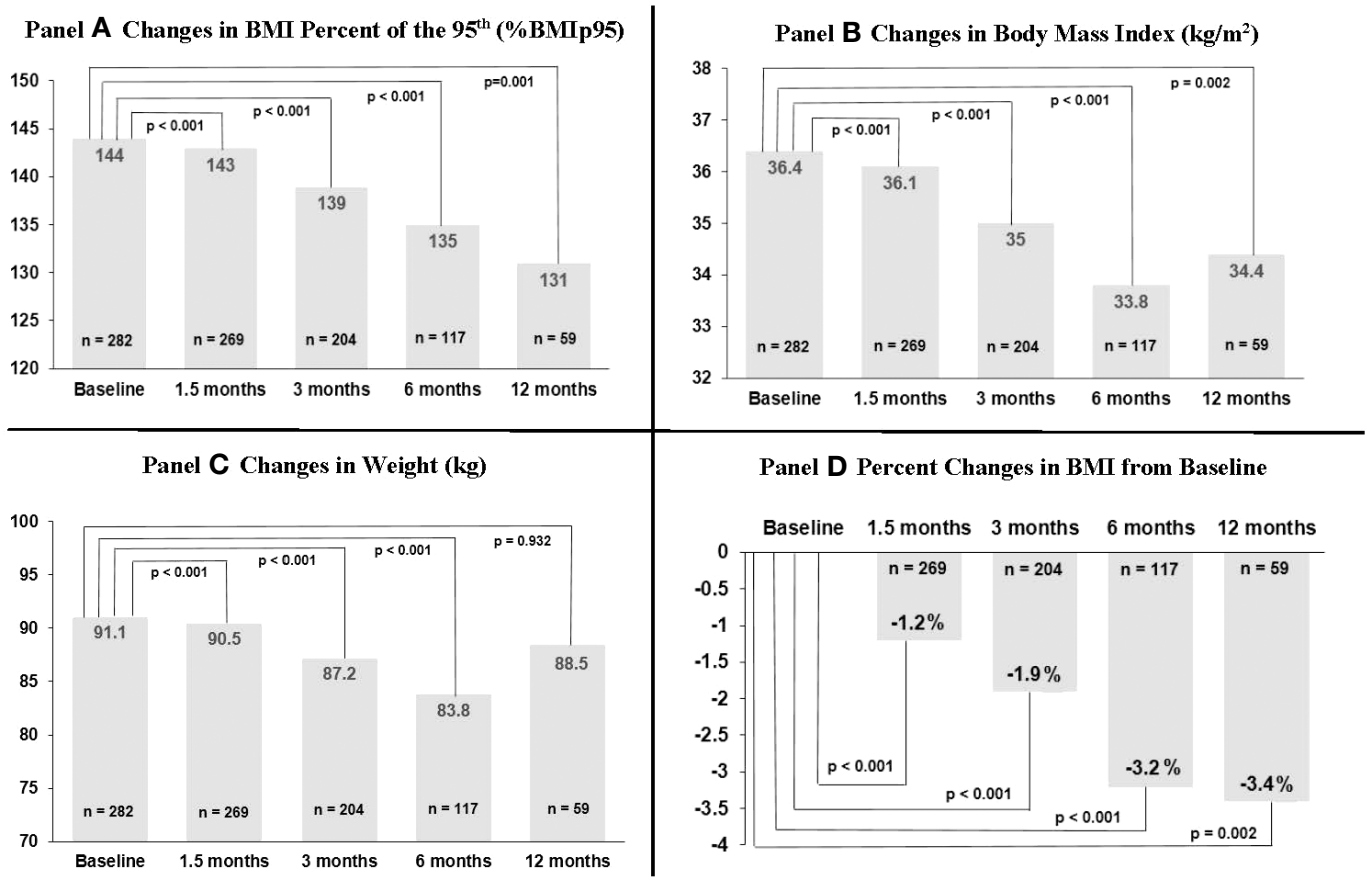
Changes in body mass index (BMI) percent of 95th percentile, BMI, and weight over time among 282 youth prescribed topiramate plus lifestyle modification in a weight management clinic.

Changes in weight-related outcomes (%BMIp95, BMI, weight) are shown in **Figure 1** and **Supplementary Table S1**. 1.5 months after topiramate initiation, %BMIp95 reduced by a mean of 2.2 percentage points (*p* < 0.001) while BMI decreased by a mean of 1.2% (absolute decrease of 0.4 kg/m^2^) among 269 patients with data available at that time point. Mean %BMIp95 continued to decrease at 3, 6, and 12 months (mean reduction in %BMIp95: 3.9, 6.6, and 9.3 percentage points, all among those with data available at those time points, respectively; all *p* ≤ 0.001 for changes over time). Absolute %BMIp95 reduced from a mean 144% at baseline to 139% at 3 months, 135% at 6 months, and 131% at 12 months, and mean BMI continued to reduce at 3, 6, and 12 months, reaching a nadir 3.4% reduction at 12 months (absolute decrease of 1.2 kg/m^2^), among those with data available at these time points.

Information on sample sizes at each time point and reasons for missing data are listed in **Supplementary Table S2**. Losses to clinic follow-up did increase over time, reaching 7.4% at 12 months. Topiramate discontinuation by provider also increased over time (7.1% among those with 3-month data available, up to 30.1% among those with 12-month data available); however, we could not ascertain reason for discontinuation in each case. Around 20%–40% of missing at each time point was due to either not having a BMI available within the analysis window, or due to being censored after additional weight-altering medications (including non-weight management related) were started or adjusted. This more commonly occurred at the 6- and 12-month time points. We additionally evaluated baseline descriptive characteristics and outcomes data among those with only data available at each time point that, overall, showed relatively minor differences, including compared to the total sample (see **Supplementary Tables S3**-**S7**). That said, reductions in %BMIp95 and BMI were overall somewhat higher in those with 12-month data available (i.e., 1.5-, 3-, and 6-month %BMIp95 reductions were 2.3, 3.6, and 5.0 percentage points among those with 6-month data available vs. 3.1, 5.6, and 9.2 percentage points among those with 12-month data available).

**Figure 2:**
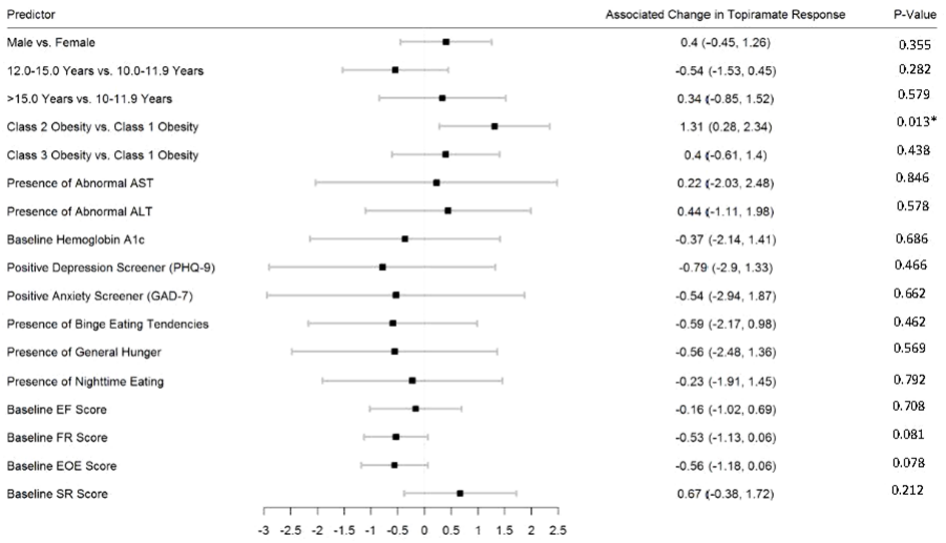
Predictors of 1.5-month change in %BMIp95. Caption: *Not statistically significant after Holm procedure for multiple comparisons (uncorrected *p*-values shown); ALT, alanine aminotransferase; AST, aspartate aminotransferase; Class 1 Obesity, body mass index percent of the 95th percentile: 100th–119th percentile; Class 2 Obesity, body mass index percent of the 95th percentile: 120th–139th percentile; Class 3 Obesity, body mass index percent of the 95th percentile: ≥140th percentile; EF, enjoyment of food; EOE, enjoyment of eating; FR, food responsiveness; GAD-7, Generalized Anxiety Disorder–7; PHQ-9, Patient Health Questionnaire–9; SR, satiety responsiveness.

**Figure 3:**
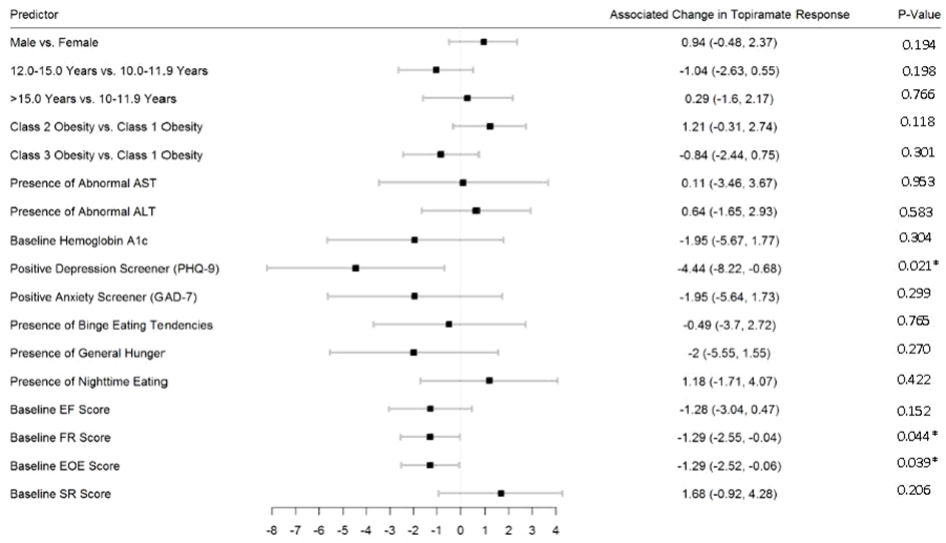
Predictors of 3-month change in %BMIp95. Caption: *Not statistically significant after Holm procedure for multiple comparisons (uncorrected *p*-values shown); ALT, alanine aminotransferase; AST, aspartate aminotransferase; Class 1 Obesity, body mass index percent of the 95th percentile: 100th–119th percentile; Class 2 Obesity, body mass index percent of the 95th percentile: 120th–139th percentile; Class 3 Obesity, body mass index percent of the 95th percentile: ≥140th percentile; EF, enjoyment of food; EOE, enjoyment of eating; FR, food responsiveness; GAD-7, Generalized Anxiety Disorder–7; PHQ-9, Patient Health Questionnaire–9; SR, satiety responsiveness.

**Figure 4:**
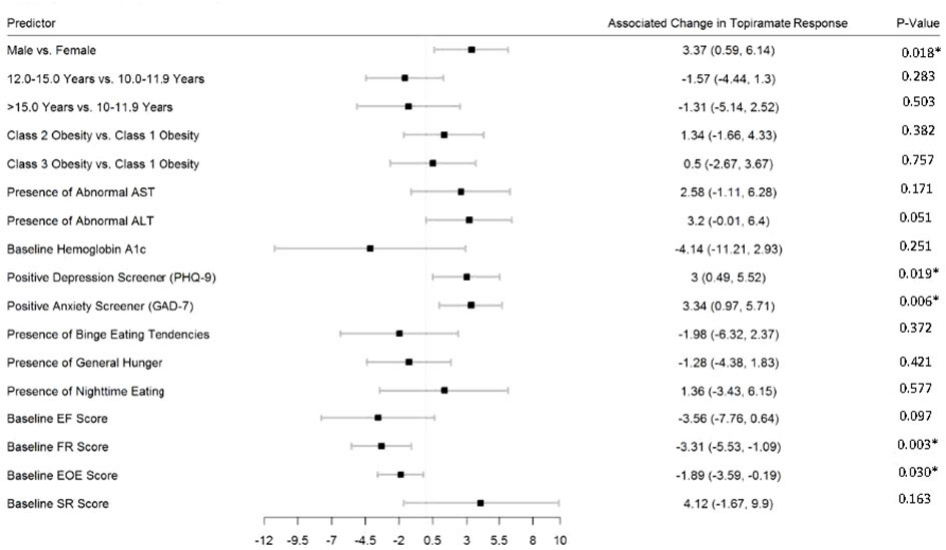
Predictors of 6-month change in %BMIp95. Caption: *Not statistically significant after Holm procedure for multiple comparisons (uncorrected *p*-values shown); ALT, alanine aminotransferase; AST, aspartate aminotransferase; Class 1 Obesity, body mass index percent of the 95th percentile: 100th–119th percentile; Class 2 Obesity, body mass index percent of the 95th percentile: 120th–139th percentile; Class 3 Obesity, body mass index percent of the 95th percentile: ≥140th percentile; EF, enjoyment of food; EOE, enjoyment of eating; FR, food responsiveness GAD-7, Generalized Anxiety Disorder–7; PHQ-9, Patient Health Questionnaire–9; SR, satiety responsiveness.

**Figure 5:**
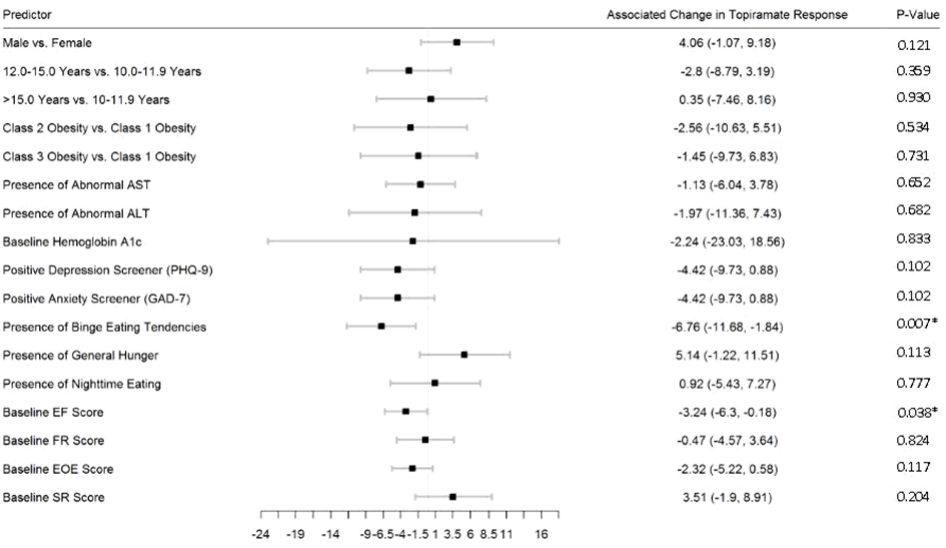
Predictors of 12-month change in %BMIp95. Caption: *Not statistically significant after Holm procedure for multiple comparisons (uncorrected *p*-values shown); ALT, alanine aminotransferase; AST, aspartate aminotransferase; Class 1 Obesity, body mass index percent of the 95th percentile: 100th–119th percentile; Class 2 Obesity, body mass index percent of the 95th percentile: 120th–139th percentile; Class 3 Obesity, body mass index percent of the 95th percentile: ≥140th percentile; EF, enjoyment of food; EOE, enjoyment of eating; FR, food responsiveness GAD-7, Generalized Anxiety Disorder–7; PHQ-9, Patient Health Questionnaire–9; SR, satiety responsiveness.

**Figures 2**–**5** show predictors of %BMIp95 change at 1.5, 3, 6, and 12 months (uncorrected *p*-values listed). After accounting for multiple comparison testing, no baseline phenotypic characteristic were statistically significantly associated with %BMIp95 reduction at any time point. However, the presence (vs. absence) of baseline depressive symptoms with 3- and 6-month, anxiety symptoms with 6-month, and binge eating tendencies with 12-month %BMIp95 changes, among others, all showed more clinically meaningful associations with topiramate plus lifestyle modification response.

## Discussion

Among youth with obesity prescribed topiramate plus lifestyle modification in a pediatric weight management clinical setting, %BMIp95 was statistically significantly reduced compared to baseline at each time point, with mean 3.9, 6.6, and 9.3 percentage point reductions at 3, 6, and 12 months, respectively, among those with data available. After accounting for multiple comparisons, no baseline phenotypic characteristics were statistically significantly associated with better or worse response.

Currently, four AOMs are U.S. FDA-approved for long-term use in youth ≥12 years old: orlistat, liraglutide 3.0 mg, phentermine/topiramate, and semaglutide 2.4 mg. That said, access to many of these medications, particularly glucagon-like peptide-1 receptor agonists (GLP1-RAs; e.g., liraglutide, semaglutide), continues to remain challenging and cost-prohibitive, and there are currently no approved options for youth <12 years old. Therefore, several others are commonly used “off-label” (e.g., phentermine in youth ≤16 years of age or for >12 weeks), and will likely continue to be used as such for the foreseeable future, with topiramate perhaps being the most common (3).

In our study, reductions in %BMIp95 and BMI from baseline remained statistically significant over the study duration in the total sample, suggesting durability and chronicity in response even up to 12 months. As a 5 percentage-point reduction in %BMIp95 has been associated with improvements in cardiometabolic risk factors, and given the natural progression for pediatric obesity to worsen over time, we consider this to be clinically relevant (56, 57). Further, our results generally align with those reported in other observational studies of youth with obesity prescribed topiramate in other (not necessarily weight management) clinical settings (19, 22, 23). For example, in a literature review (*n* = 9 studies) and case series of youth with severe obesity (*n* = 5; ages 10–11 years old; baseline %BMIp95 1.28–2.50 times the 95th percentile) prescribed topiramate, Berman et al. found that all studies reported trends toward BMI reduction, and a mean 16-week 12% reduction in %BMIp95 among those in the case series (23).

As for how our results compare to those from pediatric clinical trials, to our knowledge there has only been one trial performed evaluating topiramate plus lifestyle modification among adolescents with obesity (27). In this trial, among 16 adolescents who received topiramate plus lifestyle modification therapy, 6-month %BMIp95 reduction was 6.2 percentage points, similar to that seen among the total cohort in our study. It is important to note that this study first involved a 4-week meal replacement therapy run-in phase including shakes and pre-packaged frozen entrée meals (total caloric intake approximately 1400 kcal/day) and, therefore, findings may not be completely comparable (27).

As for clinical trials among adults, in one 6-month dose-ranging placebo-controlled trial, those receiving topiramate plus lifestyle modification therapy experienced a mean 4.8% (64 mg) to 5.0% (92 mg) weight reduction in intention-to-treat analyses (among study completers, 5.2% and 5.0% for the 64 mg and 92 mg doses, respectively) (58). In a separate longer term dose-ranging placebo-controlled trial, those receiving 92 mg topiramate plus lifestyle modification therapy experienced a mean 2.6 kg/m2 BMI reduction after approximately 1 year (18). While this is similar to that seen in our study, the former clinical trial also consisted of a 6-week placebo weight-loss run-in phase, a longer titration (8 weeks), and overall higher average doses (mean topiramate dose over the course for patients in our study ranged from 34.2 ± 20.8 to 39.7 ± 19.0 mg) (18). Further prospective cohort trials involving higher doses [i.e., often used in pediatric obesity management often range from 50–100 mg daily (27, 59)] and not including run-in interventions among children and adolescents are needed to better evaluate how response in clinical settings may compare to those in these clinical trials.

As for how these results compare with other medications, to our knowledge there have been no randomized controlled trial evaluating phentermine versus placebo (both as lifestyle modification adjuncts) among youth with obesity. One previous observational study among 25 adolescents (mean age = 16.1 years; BMI = 41.2 kg/m^2^) followed in our pediatric weight management clinic showed that adding phentermine to lifestyle modification, compared to lifestyle modification alone, resulted in a mean 4.1% greater percent change in BMI at 6 months (60). A case series among 30 children and adolescents (11–18 years old; starting BMI 31.0–51.0 kg/m^2^) prescribed phentermine plus lifestyle modifications found a mean %BMIp95 reduction of 15% (mean duration 10 months) (61).

In terms of GLP1-RAs, in a randomized controlled trial comparing liraglutide 3.0 mg with placebo (both as lifestyle modification adjuncts) on BMI reduction among 251 12 to <18 year olds with obesity (BMI ≥ 95th percentile), mean 56-week placebo-subtracted change in %BMIp95 was 6.2 percentage points (relative placebo-subtracted percent reduction in BMI 4.6%) (29). One observational study reported on seven female adolescents (mean age = 14.9 years old) with BMI ≥ 98th percentile and obesity-related complications who received liraglutide (doses ranging from 1.2–3.0 mg) in conjunction with an intense lifestyle modification program, and found that BMI reduced by a mean 2.1 kg/m^2^ after three months (62). A randomized controlled trial comparing semaglutide 2.4 mg versus placebo (both as lifestyle modifications adjuncts) among 201 12 to <18 year olds with obesity/overweight (BMI ≥95th percentile or ≥85th percentile plus ≥1 obesity-related comorbidity) found a mean 16.7% placebo-subtracted percent BMI reduction after 68 weeks (30). We are not aware of any observational studies evaluating semaglutide 2.4 mg in pediatric weight management clinical settings, likely owing to its relatively recent FDA approval. That said, one observational study among adults with obesity prescribed semaglutide showed results that were relatively comparable to those seen in a randomized controlled trial comparing semaglutide with placebo (both as lifestyle modification adjuncts) (63, 64). Overall, it appears that topiramate plus lifestyle modification response may be more aligned with that seen with phentermine and liraglutide 3.0 mg, and less than that generally observed with semaglutide 2.4 mg.

Previous studies have shown that baseline phenotypic characteristics including presence (vs. absence) of obesity, higher BMI, females (vs. males), younger age, and no/mild (vs. moderate/profound) intellectual disability have been associated with greater weight loss response over similar time points to our study (13–24). That said, these studies all involved different populations (i.e., adults or children/adults combined), doses (generally ranging from 75 to 256 mg daily), indications (epilepsy, all indications combined), and research designs (e.g., clinical trials), and therefore, results may not be comparable (13–24).

We found that, after accounting for multiple comparisons, no baseline phenotypic characteristics were statistically significantly associated with %BMIp95 reduction at any time point. That said, presence (vs. absence) of baseline depressive symptoms with 3- and 6-month, anxiety symptoms with 6-month, and binge eating tendencies with 12-month %BMIp95 changes, among others, all showed more meaningful associations with topiramate response. Most previous studies involving topiramate did not evaluate eating behaviors as potential predictors of response, with the only one to our knowledge also showing that baseline appetite was not associated with 6-month BMI response; however, appetite reduction at 3 months was predictive of 6-month BMI reduction (20). Overall, studies evaluating effects of topiramate on eating-related behaviors among youth followed in a pediatric weight management clinic are lacking. Larger scale prospective studies, including those incorporating measures of depression, anxiety, and binge eating tendencies, may help identify and confirm potential baseline characteristics associated with response that have not previously found.

### Strengths and limitations

A strength of this study is our sizable cohort of youth treated in a pediatric weight management clinic over an 8-year period. To our knowledge, this is the largest study among youth examining outcomes from topiramate plus lifestyle modification in a weight management clinical setting, and our sample size afforded us the ability to evaluate baseline phenotypic characteristics potentially associated with response to this intervention.

However, our results must be interpreted within the context of limitations. First, this was an observational study performed through EHR data review, which may be incomplete and relies on practitioners and clinical staff correctly and accurately entering all information (65). For example, among those in whom topiramate was discontinued by a provider, we could not fully ascertain the rationale (e.g., side effects, not effective, effective but potentially more effective option available [e.g., semaglutide 2.4 mg U.S. FDA-approved in 2022 and by the EMA in 2022]). The finding that those with 12-month data available had somewhat greater %BMIp95 and BMI suggests the possibility that outcomes may be biased to an extent toward greater effects. Further, clinic follow-up appointments do not occur at pre-specified times and, therefore, outcome data at various time points may be missing due to a number of potential reasons. We found that around 20%–40% of missing data at each time point was due to either not having a BMI available within the analysis window, or due to being censored after additional weight-altering medications (including non-weight management related) were started or adjusted, which was more common at the 6- and 12-month time points.

More specifically in terms of not having a BMI within the analysis window, this was due to having no follow-up within our pre-specified window for data interpolation [which could occur in those doing well on treatment (e.g., need to be seen less frequently)], virtual visits which increased significantly since COVID-19, or BMI was otherwise missing from a visit. Lost to follow-up, which has the potential to bias results in either direction and is an issue often more prevalent in observational studies compared to clinical trials, did increase over time, reaching 6.4% and 7.4% at the 6- and 12-month time points, respectively. That said, this is in-line or less than that reported in recent randomized controlled trials among adolescents evaluating phentermine/topiramate versus placebo (12-month lost to follow-up: mid-dose 16.7%, high-dose 17.7%, placebo 25%) and liraglutide versus placebo (12-month lost to follow-up: liraglutide 9.6%, placebo 16.6%), both in conjunction with lifestyle modification (28, 29).

Additionally, only around one-fifth to one-third had various elements of the clinic intake survey available for analysis. In our clinical setting, there were several potential reasons as to why intake surveys may not have been completed, including that a patient was scheduled off the clinic waitlist and did not have time to complete prior to the initial appointment, forgot to bring survey to the appointment, or survey was not entered into the database. We did find that 15% of those prescribed topiramate plus lifestyle modifications were from non-primary English language speaking families, and as surveys were only available in English until 2021 this led to higher percentages of those not completing surveys being from such families, potentially limiting generalizability. Of note, we did not find differences by insurance type (public vs. private) between those completing surveys and those not, suggesting that much of the missing intake survey data may have been due to these other factors mentioned that can occur in clinical settings.

As our EHR does not directly link to pharmacy data (e.g., to assess how often patients are refilling medications), we could not determine adherence. Moreover, we did not have measures of synthetic liver function tests (i.e., INR) or abdominal ultrasound results available (only performed as indicated in our clinic and not as part of routine care) to assess if those with presence of elevated liver transaminases had evidence of liver dysfunction and/or ultrasonographic findings suggestive of non-alcoholic fatty liver disease. Further, the precision of our estimates may have been affected by secular trends in obesity interventions stemming from COVID-19. Indeed, obesity treatments may have been less effective during this time (66). Finally, results from this study came from a single academic health center-based pediatric weight management clinic located in the Midwestern portion of the U.S. and, therefore, it is unclear how generalizable our findings are to other pediatric weight management clinics in the U.S. or worldwide.

Finally, we were unable to reliably assess for side effects given that these were not captured in our EHR systematically and consistently. Specifically, when a medication is marked as “not taking” or “discontinued,” the specific rationale may be missing, and even if the rationale is listed as “side effects,” often the specific side effect is missing as this is a free-texted element that takes time to enter in a busy clinical setting. Potential side effects reported with the use of topiramate include paresthesias, headaches, dysgeusia, dry mouth, worsening depression, and cognitive impairment, among others (59). However, these are generally dose dependent, particularly at higher doses (around 400 mg daily), while doses used for obesity management are often lower (around 50–100 mg daily) (23, 59, 67). In a Fox et al. randomized controlled trial comparing topiramate 75 mg daily with placebo following a meal-replacement run-in phase, among 30 adolescents, 25% in the topiramate group reported experiencing paresthesias versus 0% in the placebo group; however, the incidence of other side effects was generally similar between groups and there were no concerning changes in neurocognitive function (27). Further research that includes more systematic collection of side effects is needed in order to better understand their incidence with topiramate use for the treatment of obesity in a pediatric weight management clinical setting.

## Conclusions

We conclude that, among youth with obesity prescribed topiramate plus lifestyle modification in a pediatric weight management clinical setting, mean percent change in %BMIp95 and BMI were both significantly reduced at 1.5, 3, 6, and 12 months after initiation, with %BMIp95 reductions from baseline of 2.2, 3.9, 6.6, and 9.3 percentage points at these time points. After accounting for multiple comparisons, no baseline phenotypic characteristics were statistically significantly associated with better or worse response. These findings suggest that topiramate with lifestyle modification may be an effective option among youth treated in a pediatric weight management clinical setting. Future prospective studies, including clinical trials, are needed to evaluate “real-world” effectiveness and to identify and confirm potential predictors of response to topiramate plus lifestyle modification therapy in pediatric clinical settings.

## Data availability statement

The raw data supporting the conclusions of this article will be made available by the authors, without undue reservation.

## Ethics statement

The studies involving humans were approved by University of Minnesota Institutional Review Board. The studies were conducted in accordance with the local legislation and institutional requirements. Written informed consent for participation in this study was provided by the participants’ legal guardians/next of kin.

## Author contributions

EB: Conceptualization, Funding acquisition, Investigation, Methodology, Resources, Supervision, Visualization, Writing – original draft, Writing – review & editing. JC: Data curation, Formal analysis, Investigation, Methodology, Visualization, Writing – review & editing. KR: Formal analysis, Methodology, Writing – review & editing. AG: Resources, Visualization, Writing – review & editing. AK: Methodology, Resources, Visualization, Writing – review & editing. CF: Methodology, Resources, Visualization, Writing – review & editing.
