# Supplementary material for: Clinical effectiveness and predictors of response to topiramate plus lifestyle modification in youth with obesity seen in a weight management clinical setting
Source: Front Endocrinol (Lausanne). 2024 May 10;15:1369270. doi: 10.3389/fendo.2024.1369270 (PMC11116594; doi:10.3389/fendo.2024.1369270)
Supplement: Supplementary file 1 [file Table_1.docx]

Supplementary Material

**Clinical Effectiveness and Predictors of Response to Topiramate plus Lifestyle Modification in Youth with Obesity seen in a Weight Management Clinical Setting**

Eric M Bomberg*, Justin Clark, Kyle D Rudser, Amy C Gross, Aaron S Kelly, Claudia K Fox, MD, MPH

*****Correspondence**:** Eric Morris Bomberg: [bombe002@umn.edu](mailto:bombe002@umn.edu).

**Supplementary Table 1:** Body mass index (BMI) percent of the 95^th^ percentile, BMI, and weight changes 1.5, 3, 6, and 12 months after starting topiramate plus lifestyle modification therapy (total sample)

|  | Baseline | 1.5 months | p-value | 3 months | p-value | 6 months | p-value | 12 months | p-value |
| --- | --- | --- | --- | --- | --- | --- | --- | --- | --- |
| n | 282 | 269 | - | 204 | - | 117 | - | 59 | - |
| Topiramate Dose^a^ (mean, SD) | - | 34.2 ± 20.8 | N/A | 34.4 ± 20.1 | N/A | 36.4 ± 19.8 | N/A | 39.7 ± 19.0 | N/A |
| Absolute Values Over Time (mean, SD) | | | | | | | | | |
| %BMIp95 | 144 ± 26 | 143 ± 27 | N/A^a^ | 139 ± 26 | N/A^a^ | 135 ± 28 | N/A^a^ | 131 ± 27 | N/A^a^ |
| BMI (kg/m^2^) | 36.4 ± 7.6 | 36.1 ± 7.8 | N/A^a^ | 35.0 ± 7.2 | N/A^a^ | 33.8 ± 7.2 | N/A^a^ | 34.4 ± 8.0 | N/A^a^ |
| Weight (kg) | 91.1 ± 28.9 | 90.5 ± 29.0 | N/A^a^ | 87.2 ± 27.9 | N/A^a^ | 83.8 ± 26.1 | N/A^a^ | 88.5 ± 25.9 | N/A^a^ |
| Changes Over Time (mean, 95% CI)^b^ | | | | | | | | | |
| %BMIp95 | N/A | -2.2 (-2.6, -1.8) | <0.001 | -3.9 (-4.5, -3.2) | <0.001 | -6.6 (-7.9, -5.2) | <0.001 | -9.3 (-12.0, -6.7) | <0.001 |
| BMI (kg/m^2^; % change) | N/A | -1.2 (-1.4, -0.9) | <0.001 | -1.9 (-2.4, -1.4) | <0.001 | -3.2 (-4.1, -2.2) | <0.001 | -3.4 (-5.4, -1.3) | 0.002 |
| BMI (kg/m^2^; absolute change) | N/A | -0.4 (-0.5, -0.3) | <0.001 | -0.7 (-0.9, -0.5) | <0.001 | -1.1 (-1.5, -0.8) | <0.001 | -1.2 (-1.9, -0.5) | 0.001 |
| Weight (kg) | N/A | -0.7 (-0.9, -0.4) | <0.001 | -1.0 (-1.4, -0.6) | <0.001 | -1.2 (-2.1, -0.3) | <0.001 | -0.1 (-2.1, 1.9) | 0.932 |
| p-values are change from baseline measures; BMI = body mass index, %BMIp95 = BMI percent of the 95^th^ percentile, SD = standard deviation  ^a^ Average over entire course among patients whose data went out to that time point  ^b^ N/A = p-values computed to assess statistical significance of changes over time from baseline and not directly relevant to means and SDs at a given time point  ^c^ Weight and %BMIp95 presented as absolute changes, while BMI presented as percent changes | | | | | | | | | |

**Supplemental Table 2:** Sample sizes and reasons for missing data at each time point for 282 youth prescribed topiramate plus lifestyle modifications in a weight management clinic (February 2012 – December 2020)

|  | **Baseline** | **1.5 months** | **3 months** | **6 months** | **12 months** |
| --- | --- | --- | --- | --- | --- |
| Lost to follow-up | - | 0 | 6 | 18 | 21 |
| No follow-up BMI data within analysis window^a^ (due to either no follow-up within window or virtual visit; therefore, no height and/or weight available), or BMI otherwise data missing at encounter | - | 13 | 30 | 40 | 37 |
| Discontinued by provider (i.e., not effective, side effect, different stimulant medication started) | - | 0 | 20 | 53 | 87 |
| Additional anti-obesity medication(s)^b^ and/or weight-altering medication(s)^c^ started or doses adjusted during analysis window (phentermine still continued) | - | 0 | 22 | 54 | 78 |
| # Analyzed at time point | 282 | 269 | 204 | 117 | 59 |
| ^a^ Weight-related outcomes determined within the following windows: 1.5 months (any visit 4-8 weeks after initial prescription), 3 months (any visit 9-15 weeks), 6 months (any visit 20-28 weeks), 12 months (any visit 44-60 weeks)  ^b^ Including glucagon-like peptide-1 receptor agonists (i.e. liraglutide, semaglutide), metformin, naltrexone ± bupropion, orlistat, and phentermine. As these can impact body mass index, data from patients starting additional anti-obesity medications between time points were censored (excluded from analyses) at and after that time point  ^c^ Including atypical antipsychotics, stimulants to treat attention-deficit hyperactivity disorder, insulin, and oral glucocorticoids. As these can impact body mass index, data from patients starting or altering additional weight altering medications between time points were censored (excluded from analyses) at and after that time point | | | | | |

**Supplemental Table 3:** Baseline descriptive statistics for 269 youth prescribed topiramate plus lifestyle modification therapy in a weight management clinic with 1.5 month data available (February 2012 – December 2020)

|  | # Patients with Data Available | Value |
| --- | --- | --- |
| Sex | | |
| Female (n, %) | 269 | 165 (61%) |
| Anthropometrics | | |
| Age, years (mean, SD) | 269 | 12.7 ± 3.1 |
| Weight, kg (mean, SD) | 269 | 91.2 ± 29.2 |
| BMI, kg/m^2^ (mean, SD) | 269 | 36.6 ± 7.7 |
| %BMIp95 (mean, SD) | 269 | 145 ± 26 |
| Insurance Type | | |
| Public | 214 | 102 (48%) |
| Private | 214 | 112 (52%) |
| Liver Function Tests: AST | | |
| Liver function: AST (mean, SD) | 115 | 28.4 ± 26.9 |
| Presence of abnormal AST based on lab value (n, %) | 115 | 22 (18%) |
| Liver Function Tests: ALT | | |
| Liver function: ALT (mean, SD) | 121 | 42.0 ± 51.5 |
| Presence of abnormal ALT based on lab value (n, %) | 121 | 11 (10%) |
| Glycemic Status |  |  |
| Hemoglobin A1c (mean, SD) | 121 | 5.4 ± 0.4 |
| Eating Behaviors and Co-Morbid Psychiatric Diagnoses | | |
| Presence of binge eating tendencies (n, %) | 50 | 16 (32%) |
| Presence of general hunger (n, %) | 49 | 34 (69%) |
| Presence of nighttime eating (n, %) | 50 | 17 (34%) |
| Met criteria for depressive symptoms^a^ (n, %) | 47 | 12 (26%) |
| Met criteria for anxiety symptoms^b^ (n, %) | 47 | 9 (19%) |
| Childhood Eating Behavior Questionnaire Scores | | |
| Food Responsiveness (mean, SD) | 85 | 3.5 ± 1.2 |
| Emotional Over-Eating (mean, SD) | 87 | 2.8 ± 1.0 |
| Enjoyment of Food (mean, SD) | 86 | 4.1 ± 0.8 |
| Satiety Responsiveness (mean, SD) | 85 | 2.1 ± 0.6 |
| ALT = alanine aminotransferase; AST = Aspartate aminotransferase; BMI = body mass index; %BMIp95 = BMI percent of the 95^th^ percentile; Reference Ranges AST: 3-11 years old, 0-50 mg/dl; 12-19 years old, 0-35 mg/dl; ≥ 20 years old: 0-45 mg/dl); Reference Ranges ALT: 0-19 years old, 0-50 mg/dl; ≥ 20 years old male, 0-70 mg/dl; ≥ 20 years old female, 0-50 mg/dl; ^a^ Met criteria for depression based on Patient Health Questionnaire-9 score ≥5 indicating mild depression or higher ; ^b^ Met criteria for anxiety based on Generalized Anxiety Disorder-7 score ≥ 10 indicating moderate or severe anxiety; ^c^ Childhood Eating Behavior Questionnaire Scores: items score 1-5 (higher scores indicate higher intensity of specific eating behavior) | | |

**Supplemental Table 4:** Baseline descriptive statistics for 204 youth prescribed topiramate plus lifestyle modification therapy in a weight management clinic with 3 month data available (February 2012 –December 2020)

|  | # Patients with Data Available | Value |
| --- | --- | --- |
| Sex | | |
| Female (n, %) | 204 | 126 (62%) |
| Anthropometrics | | |
| Age, years (mean, SD) | 204 | 12.5 ± 3.1 |
| Weight, kg (mean, SD) | 204 | 88.2 ± 28.7 |
| BMI, kg/m^2^ (mean, SD) | 204 | 35.7 ± 7.3 |
| %BMIp95 (mean, SD) | 204 | 143 ± 25 |
| Insurance Type | | |
| Public | 161 | 80 (50%) |
| Private | 161 | 81(50%) |
| Liver Function Tests: AST | | |
| Liver function: AST (mean, SD) | 87 | 29.8 ± 30.2 |
| Presence of abnormal AST based on lab value (n, %) | 87 | 8 (9%) |
| Liver Function Tests: ALT | | |
| Liver function: ALT (mean, SD) | 92 | 43.1 ± 57.2 |
| Presence of abnormal ALT based on lab value (n, %) | 92 | 17 (18%) |
| Glycemic Status |  |  |
| Hemoglobin A1c (mean, SD) | 93 | 5.4 ± 0.3 |
| Eating Behaviors and Co-Morbid Psychiatric Diagnoses | | |
| Presence of binge eating tendencies (n, %) | 29 | 12 (41%) |
| Presence of general hunger (n, %) | 29 | 22 (76%) |
| Presence of nighttime eating (n, %) | 29 | 10 (34%) |
| Met criteria for depressive symptoms^a^ (n, %) | 26 | 7 (27%) |
| Met criteria for anxiety symptoms^b^ (n, %) | 27 | 4 (15%) |
| Childhood Eating Behavior Questionnaire Scores | | |
| Food Responsiveness (mean, SD) | 59 | 3.7 ± 1.1 |
| Emotional Over-Eating (mean, SD) | 60 | 2.8 ± 1.0 |
| Enjoyment of Food (mean, SD) | 59 | 4.3 ± 0.8 |
| Satiety Responsiveness (mean, SD) | 60 | 2.1 ± 0.6 |
| ALT = alanine aminotransferase; AST = Aspartate aminotransferase; BMI = body mass index; %BMIp95 = BMI percent of the 95^th^ percentile; Reference Ranges AST: 3-11 years old, 0-50 mg/dl; 12-19 years old, 0-35 mg/dl; ≥ 20 years old: 0-45 mg/dl); Reference Ranges ALT: 0-19 years old, 0-50 mg/dl; ≥ 20 years old male, 0-70 mg/dl; ≥ 20 years old female, 0-50 mg/dl; ^a^ Met criteria for depression based on Patient Health Questionnaire-9 score ≥5 indicating mild depression or higher ; ^b^ Met criteria for anxiety based on Generalized Anxiety Disorder-7 score ≥ 10 indicating moderate or severe anxiety; ^c^ Childhood Eating Behavior Questionnaire Scores: items score 1-5 (higher scores indicate higher intensity of specific eating behavior) | | |

**Supplemental Table 5:** Baseline descriptive statistics for 117 youth prescribed topiramate plus lifestyle modification therapy in a weight management clinic with 6 month data available (February 2012 – December 2020)

|  | # Patients with Data Available | Value |
| --- | --- | --- |
| Sex | | |
| Female (n, %) | 117 | 68 (58%) |
| Anthropometrics | | |
| Age, years (mean, SD) | 117 | 12.1 ± 2.9 |
| Weight, kg (mean, SD) | 117 | 85.1 ± 27.6 |
| BMI, kg/m^2^ (mean, SD) | 117 | 35.0 ± 7.2 |
| %BMIp95 (mean, SD) | 117 | 142 ± 26 |
| Insurance Type | | |
| Public | 98 | 43 (44%) |
| Private | 98 | 55 (56%) |
| Liver Function Tests: AST | | |
| Liver function: AST (mean, SD) | 51 | 32.4 ± 25.5 |
| Presence of abnormal AST based on lab value (n, %) | 51 | 6 (12%) |
| Liver Function Tests: ALT | | |
| Liver function: ALT (mean, SD) | 53 | 48.0 ± 51.8 |
| Presence of abnormal ALT based on lab value (n, %) | 53 | 13 (25%) |
| Glycemic Status |  |  |
| Hemoglobin A1c (mean, SD) | 54 | 5.5 ± 0.3 |
| Eating Behaviors and Co-Morbid Psychiatric Diagnoses | | |
| Presence of binge eating tendencies (n, %) | 13 | 4 (31%) |
| Presence of general hunger (n, %) | 13 | 9 (69%) |
| Presence of nighttime eating (n, %) | 13 | 4 (31%) |
| Met criteria for depressive symptoms^a^ (n, %) | 11 | 1 (9%) |
| Met criteria for anxiety symptoms^b^ (n, %) | 12 | 1 (8%) |
| Childhood Eating Behavior Questionnaire Scores | | |
| Food Responsiveness (mean, SD) | 34 | 3.7 ± 1.2 |
| Emotional Over-Eating (mean, SD) | 35 | 2.5 ± 1.1 |
| Enjoyment of Food (mean, SD) | 34 | 4.2 ± 0.8 |
| Satiety Responsiveness (mean, SD) | 35 | 2.0 ± 0.6 |
| ALT = alanine aminotransferase; AST = Aspartate aminotransferase; BMI = body mass index; %BMIp95 = BMI percent of the 95^th^ percentile; Reference Ranges AST: 3-11 years old, 0-50 mg/dl; 12-19 years old, 0-35 mg/dl; ≥ 20 years old: 0-45 mg/dl); Reference Ranges ALT: 0-19 years old, 0-50 mg/dl; ≥ 20 years old male, 0-70 mg/dl; ≥ 20 years old female, 0-50 mg/dl; ^a^ Met criteria for depression based on Patient Health Questionnaire-9 score ≥5 indicating mild depression or higher ; ^b^ Met criteria for anxiety based on Generalized Anxiety Disorder-7 score ≥ 10 indicating moderate or severe anxiety; ^c^ Childhood Eating Behavior Questionnaire Scores: items score 1-5 (higher scores indicate higher intensity of specific eating behavior) | | |

**Supplemental Table 6:** Baseline descriptive statistics for 59 youth prescribed topiramate plus lifestyle modification therapy in a weight management clinic with 12 month data available (February 2012 –December 2020)

|  | # Patients with Data Available | Value |
| --- | --- | --- |
| Sex | | |
| Female (n, %) | 59 | 37 (63%) |
| Anthropometrics | | |
| Age, years (mean, SD) | 59 | 12.8 ± 2.6 |
| Weight, kg (mean, SD) | 59 | 88.5 ± 27.6 |
| BMI, kg/m^2^ (mean, SD) | 59 | 35.6 ± 7.9 |
| %BMIp95 (mean, SD) | 59 | 140 ± 26 |
| Insurance Type | | |
| Public | 51 | 24 (47%) |
| Private | 51 | 27 (53%) |
| Liver Function Tests: AST | | |
| Liver function: AST (mean, SD) | 25 | 25.0 ± 13.0 |
| Presence of abnormal AST based on lab value (n, %) | 25 | 2 (8%) |
| Liver Function Tests: ALT | | |
| Liver function: ALT (mean, SD) | 25 | 34.6 ± 35.7 |
| Presence of abnormal ALT based on lab value (n, %) | 25 | 3 (12%) |
| Glycemic Status |  |  |
| Hemoglobin A1c (mean, SD) | 26 | 5.4 ± 0.3 |
| Eating Behaviors and Co-Morbid Psychiatric Diagnoses | | |
| Presence of binge eating tendencies (n, %) | 11 | 2 (18%) |
| Presence of general hunger (n, %) | 11 | 7 (64%) |
| Presence of nighttime eating (n, %) | 11 | 3 (27%) |
| Met criteria for depressive symptoms^a^ (n, %) | 9 | 1 (11%) |
| Met criteria for anxiety symptoms^b^ (n, %) | 9 | 1 (11%) |
| Childhood Eating Behavior Questionnaire Scores | | |
| Food Responsiveness (mean, SD) | 16 | 3.8 ± 0.9 |
| Emotional Over-Eating (mean, SD) | 17 | 2.5 ± 1.0 |
| Enjoyment of Food (mean, SD) | 16 | 4.3 ± 0.8 |
| Satiety Responsiveness (mean, SD) | 17 | 2.0 ± 0.5 |
| ALT = alanine aminotransferase; AST = Aspartate aminotransferase; BMI = body mass index; %BMIp95 = BMI percent of the 95^th^ percentile; Reference Ranges AST: 3-11 years old, 0-50 mg/dl; 12-19 years old, 0-35 mg/dl; ≥ 20 years old: 0-45 mg/dl); Reference Ranges ALT: 0-19 years old, 0-50 mg/dl; ≥ 20 years old male, 0-70 mg/dl; ≥ 20 years old female, 0-50 mg/dl; ^a^ Met criteria for depression based on Patient Health Questionnaire-9 score ≥5 indicating mild depression or higher ; ^b^ Met criteria for anxiety based on Generalized Anxiety Disorder-7 score ≥ 10 indicating moderate or severe anxiety; ^c^ Childhood Eating Behavior Questionnaire Scores: items score 1-5 (higher scores indicate higher intensity of specific eating behavior) | | |

**Supplementary Table 7:** Mean weight, body mass index, and percent of the 95^th^ body mass index percentile 1.5, 3, 6, and 12 months after starting topiramate plus lifestyle modification therapy (among those with data available at time points)

| 1.5 Month Data Available | | | | | | | | | |
| --- | --- | --- | --- | --- | --- | --- | --- | --- | --- |
| n=269 | **Baseline** | **1.5 months** | **p-value** | **3 months** | **p-value** | **6 months** | **p-value** | **12 months** | **p-value** |
| Absolute Values Over Time (mean, SD) | | | | | | | | | |
| %BMIp95 | 153 ± 29 | 151 ± 30 | N/A^a^ | - | - | - | - | - | - |
| BMI (kg/m^2^) | 39.7 ±8.6 | 39.4 ± 8.9 | N/A^a^ | - | - | - | - | - | - |
| Weight (kg) | 102.1 ±29.7 | 101.8 ± 29.9 | N/A^a^ | - | - | - | - | - | - |
| Changes Over Time (mean, 95% CI)^b^ | | | | | | | | | |
| %BMIp95 | N/A | -1.8 | <0.001 | - | - | - | - | - | - |
| %BMI (kg/m^2^) | N/A | -0.9 | 0.01 | - | - | - | - | - | - |
| Weight (kg) | N/A | -0.3 | 0.27 | - | - | - | - | - | - |
| 3 Month Data Available | | | | | | | | | |
| n=204 | **Baseline** | **1.5 months** | **p-value** | **3 months** | **p-value** | **6 months** | **p-value** | **12 months** | **p-value** |
| Absolute Values Over Time (mean, SD) | | | | | | | | | |
| %BMIp95 | 142 ± 25 | 141 ± 25 | N/A^a^ | 139 ± 24 | N/A^a^ | - | - | - | - |
| BMI (kg/m^2^) | 36.2 ± 7.1 | 35.9 ± 7.1 | N/A^a^ | 35.7 ± 7.1 | N/A^a^ | - | - | - | - |
| Weight (kg) | 91.2 ± 29.1 | 90.7 ± 28.8 | N/A^a^ | 90.8 ± 28.8 | N/A^a^ | - | - | - | - |
| Changes Over Time (mean, 95% CI)^b^ | | | | | | | | | |
| %BMIp95 | N/A | -1.9 | < 0.001 | -3.1 | <0.001 | - | - | - | - |
| %BMI (kg/m^2^) | N/A | -0.9 | < 0.001 | -1.4 | <0.001 | - | - | - | - |
| Weight (kg) | N/A | -0.5 | 0.01 | -0.4 | 0.16 | - | - | - | - |
| 6 Month Data Available | | | | | | | | | |
| n=117 | **Baseline** | **1.5 months** | **p-value** | **3 months** | **p-value** | **6 months** | **p-value** | **12 months** | **p-value** |
| Absolute Values Over Time (mean, SD) | | | | | | | | | |
| %BMIp95 | 144 ± 25 | 143 ± 26 | N/A^a^ | 142 ± 26 | N/A^a^ | 139 ± 27 | N/A^a^ | - | - |
| BMI (kg/m^2^) | 34.8 ± 6.5 | 34.4 ± 6.6 | N/A^a^ | 34.2 ± 6.7 | N/A^a^ | 34.1 ± 6.5 | N/A^a^ | - | - |
| Weight (kg) | 84.2 ± 27.2 | 82.3 ± 26.7 | N/A^a^ | 81.8 ± 26.3 | N/A^a^ | 83.9 ± 25.5 | N/A^a^ | - | - |
| Changes Over Time (mean, 95% CI)^b^ | | | | | | | | | |
| %BMIp95 | N/A | -2.3 | <0.001 | -3.6 | <0.001 | -5.0 | <0.001 | - | - |
| %BMI (kg/m^2^) | N/A | -1.2 | <0.001 | -1.7 | <0.001 | -2.0 | <0.001 | - | - |
| Weight (kg) | N/A | -0.8 | 0.01 | -0.9 | 0.03 | -0.3 | 0.59 | - | - |
| 12 Month Data Available | | | | | | | | | |
| n=59 | **Baseline** | **1.5 months** | **p-value** | **3 months** | **p-value** | **6 months** | **p-value** | **12 months** | **p-value** |
| Absolute Values Over Time (mean, SD) | | | | | | | | | |
| %BMIp95 | 140 ± 26 | 137 ± 26 | N/A^a^ | 135 ± 28 | N/A^a^ | 130 ± 28 | N/A^a^ | 131 ± 27 | N/A^a^ |
| BMI (kg/m^2^) | 35.7 ± 7.9 | 35.2 ± 8.1 | N/A^a^ | 34.6 ± 8.1 | N/A^a^ | 33.3 ± 8.1 | N/A^a^ | 34.4 ± 8.0 | N/A^a^ |
| Weight (kg) | 88.5 ± 27.6 | 87.9 ± 28.0 | N/A^a^ | 87.0 ± 27.6 | N/A^a^ | 83.7 ± 27.3 | N/A^a^ | 88.5 ± 25.9 | N/A^a^ |
| Changes Over Time (mean, 95% CI)^b^ | | | | | | | | | |
| %BMIp95 | N/A | -3.1 | <0.001 | -5.6 | <0.001 | -9.2 | <0.001 | -9.3 | <0.001 |
| %BMI (kg/m^2^) | N/A | -1.8 | <0.001 | -3.2 | <0.001 | -5.1 | <0.001 | -3.4 | <0.001 |
| Weight (kg) | N/A | -1.2 | <0.001 | -2.1 | <0.001 | -2.7 | <0.001 | -0.1 | 0.93 |
| p-values are change from baseline measures; BMI = body mass index, %BMIp95 = BMI percent of the 95^th^ percentile, SD = standard deviation  ^a^ N/A = p-values computed to assess statistical significance of changes over time and are not directly relevant to means and SDs at a given time point  ^b^ Weight and %BMIp95 are presented as absolute changes, while BMI is presented as percent changes | | | | | | | | | |
